# Chronic anemia complicated by cardiac failure, pulmonary hypertension, and pericardial effusion: a case report

**DOI:** 10.1186/s13256-022-03686-z

**Published:** 2023-02-07

**Authors:** Muhammad Yousaf, Memon iIlahi, Aisha Bibi, Hadeel Elhassan, Muhammad Sharif, Abdul Rehman Abid, Maya Ali Omran, Arwa Hassan, Khawaja Hassan Haroon

**Affiliations:** 1 Hazm Mebaireek Hospital, Hamad Medical Corporation, Doha, Qatar; 2 Weill Cornell Medicine Qatar, Cornell University, Doha, Qatar

**Keywords:** Anemia, Iron deficiency, Pulmonary hypertension, Cardiac failure, Pericardial effusion, Heart failure

## Abstract

**Background:**

Worldwide, iron deficiency anaemia (IDA) is the most common cause of anaemia. Iron deficiency alone has an association with heart failure and pulmonary hypertension. Chronic iron deficiency anemia triggers various physiologic adjustments, leading to hyperdynamic circulation and enhanced hypoxic pulmonary vasoconstriction. Those mechanisms may result in the development of high output cardiac failure and pulmonary hypertension; however, pericardial effusion remains a rare association.

**Case presentation:**

A 44-year-old Nepalese man presented with fatigability and swollen ankles. Except for a hemorrhoidectomy 4 years ago, he had no comorbidities. Labs confirmed severe iron deficiency anemia (hemoglobin 1.8 grams per deciliter) likely secondary to hemorrhoids. An echocardiogram revealed high output cardiac failure, pericardial effusion, and severe pulmonary hypertension. He responded well to the correction of anemia and diuretics with the resolution of vascular complications.

**Conclusion:**

We report a unique presentation of chronic severe iron deficiency anemia complicated by heart failure, pulmonary hypertension, and pericardial effusion. We believe it to be the first-ever such case reported in the literature. These cardiovascular complications seem to result from internal homeostatic mechanisms against the chronic tissue hypoxemia observed in severe anemia. Furthermore, iron deficiency alone has an association with heart failure and pulmonary hypertension. After excluding other potential causes, we confirmed iron deficiency anaemia as the cause of those complications. The correction of anemia led to an excellent recovery without any sequelae. Our case report highlights the fact that management of such a case should be focused on underlying etiology rather than the complications.

## Case report

### Background

Anemia affects a quarter of the world’s population, while iron deficiency anemia (IDA) is the predominant cause of anemia across the globe [1]. Iron deficiency, even without overt anemia, has an association with heart failure (HF) and pulmonary hypertension (PH) [2]. While IDA complicated by pericardial effusion alone is rare [3], we report a presentation of chronic IDA complicated by cardiac failure, pulmonary hypertension, and pericardial effusion. To our knowledge, this is a rare presentation of IDA, never reported in the literature. Based on available scientific evidence, we review the pathophysiology and therapeutic implications of this rare presentation.

### Case report

A 44-year-old Nepalese man was referred to the emergency department for the management of severe anemia. He had an unremarkable medical history except for a hemorrhoidectomy 4 years ago. He was not taking any regular medications. A mason by profession, he never had alcohol or smoked cigarettes but occasionally chewed tobacco. He presented with fatigability and swollen ankles for 10 days. There was no history of fever, night sweats, or weight loss. He did not report cough, shortness of breath, chest pain, or palpitations. There was no melena except for a single episode of a small amount of fresh blood per rectum 2 days ago.

On admission, his vitals were a blood pressure of 120/56 mmHg, pulse 86 beats per minute, respiratory rate 19 breaths per minute, temperature 36.7 °C, and SpO_2_ 100% on air. On exam, he had marked pallor of skin and conjunctiva, clubbing, and mild bilateral pedal edema. Heart sounds appeared normal with a systolic murmur at the tricuspid area. There was no lymphadenopathy, and the rest of the examination was unremarkable.

On admission, he had extreme microcytic anemia with a hemoglobin (Hb) of 1.8 g/dL (normal range 13–17 g/dL) with a mean corpuscular volume (MCV) of 58 fl (normal range 83–101 fL), and hematocrit (Hct) of 8% (normal range 40–50%). Other labs revealed a normal white blood cell (WBC) and platelet count, C-reactive protein (CRP), haptoglobin, and liver and renal function tests. Hematinic confirmed iron deficiency anemia (IDA) with ferritin 4.2 μg/L (normal range 30–490 μg/L), serum iron 2 μmol/L (normal range 6–35 μmol/L), total iron binding capacity (TIBC) 93 μmol/L (normal range 45–80 μmol/L), transferrin 3.7 g/L (normal range 2–3.6 g/L), iron saturation 2% (normal range 15–45%). He had a low reticulocyte count of 36 × 10^3^/μL (normal range 50–100 × 10^3^/μL). A blood film showed a severe hypochromic microcytic picture with anisopoikilocytosis, rouleaux, dimorphism, few target cells, and teardrops.

On proctoscopy, he had a small internal hemorrhoid with no active bleeding. The stool for ova and parasites came back as negative. The guaiac fecal occult blood test was positive. The remaining extensive workup was noncontributory, including B12, folate, anti-transglutaminase immunoglobulin G (IgG) antibody (Ab), hemoglobin electrophoresis, and a computed tomography (CT) scan of the abdomen and pelvis.

**Fig. 1:**
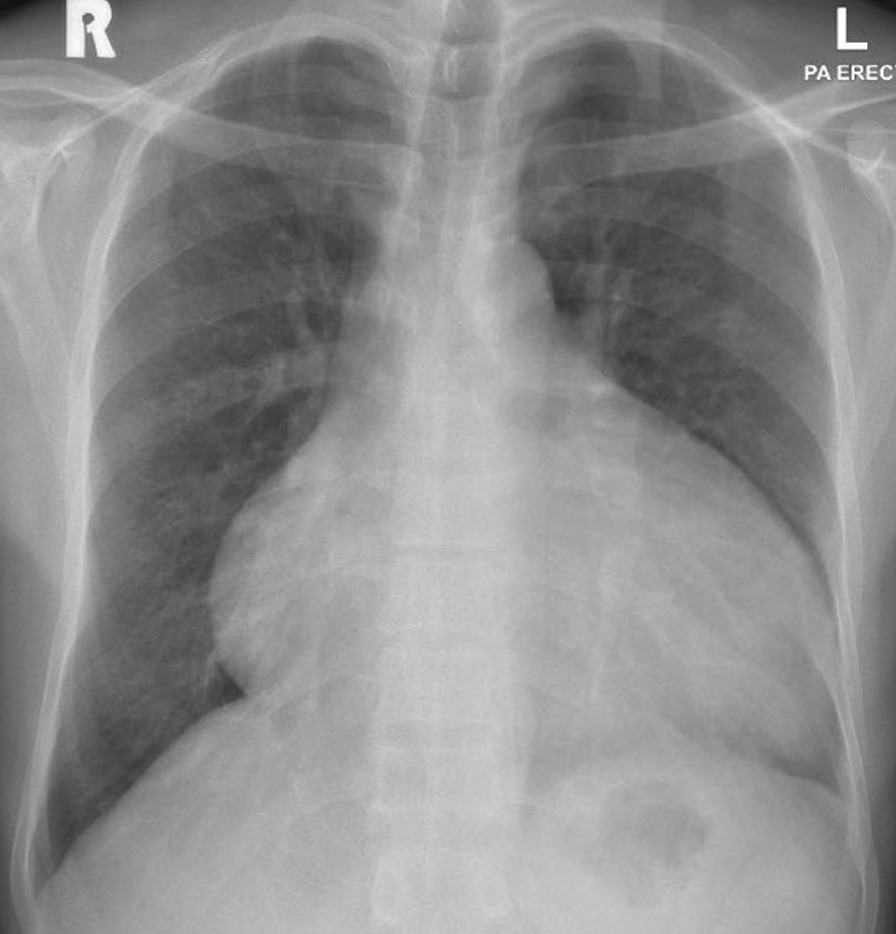
A chest X-ray on admission showed cardiomegaly and pericardial effusion

There was cardiomegaly on the chest X-ray (Fig. 1). An echocardiogram, on admission, showed high output cardiac failure [cardiac index 6.77 L/minute/m^2^) with mild dilatation and reduction in left ventricular (LV) function (ejection fraction (EF) 41%] and moderate pericardial effusion (no tamponade). Thyroid functions were unremarkable. The echocardiogram also showed severely raised pulmonary artery systolic pressure (PASP, 80 mmHg) with moderate tricuspid regurgitation and a tricuspid annular plane systolic excursion (TAPSE) of 2.2 cm.

The above findings led to a diagnosis of chronic severe iron deficiency anemia (IDA) complicated by high output cardiac failure, pulmonary hypertension, and pericardial effusion. The case was managed with blood transfusion and diuretics, furosemide 40 mg daily for 1 week. He received four units of packed red blood cells (RBC), raising his hemoglobin from Hb 1.8 to Hb 8.5 within 24 hours. Iron stores were replenished with intravenous iron infusion followed by oral iron as an outpatient.

**Fig. 2:**
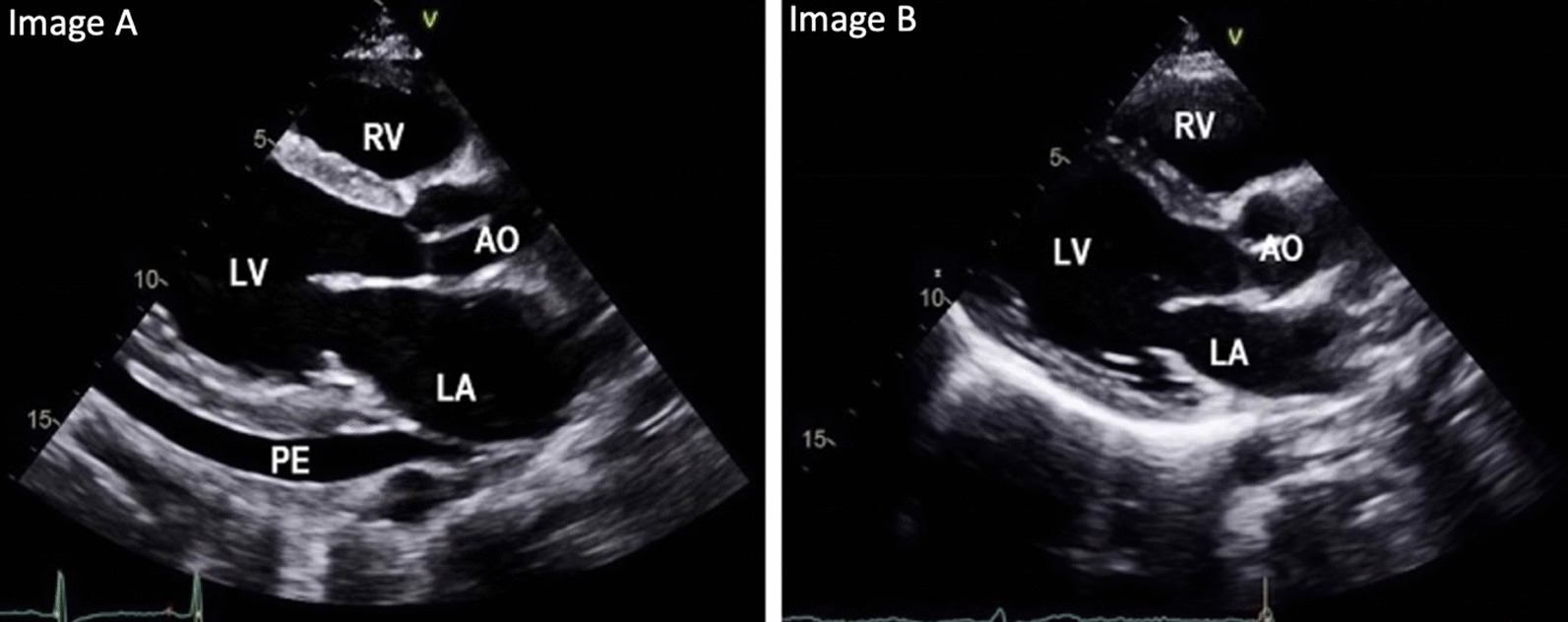
Echocardiogram at admission showing pericardial effusion (PE) (**A**). Repeat echo after treatment showing resolution of pericardial effusion (**B**)

Clinically, patient responded well to the correction of anemia (Hb 12.4 with MCV 88) with the resolution of fatigue and pedal edema. A repeat echocardiogram a week later showed a reduction of PASP (35 mmHg) and a decrease in LV stroke volume and cardiac output and resolution in the pericardial effusion (Fig. 2). The patient was discharged home with follow-up in a general medicine clinic. The discharge medications included oral iron only. On follow-up at 4 months, his Hb remained stable at Hb 15.5 and MCV 86. A repeat echocardiogram showed resolution of PH (PASP, 29 mmHg) and normalization of the cardiac index to 2.1 L/minute/m^2^.

## Discussion

We report a case of chronic severe iron deficiency anemia (IDA) presenting with heart failure (HF), pulmonary hypertension (PH), and pericardial effusion. These distinct cardiovascular pathologies caused by chronic severe iron deficiency anemia seem to be interlinked with a unified pathophysiology as reviewed below in the light literature. We have summarized key physiologic changes observed in severe anemia in Table 1.

**Table 1:** Pathophysiology adjustments in chronic severe anemia

Pathophysiology adjustments in chronic severe anemia	
1. *Low blood viscosity* due to low number of circulating red cells 2. *Peripheral vasodilation* via enhanced nitric oxide–mediated vasodilation 3. *Salt and fluid retention* (neurohumoral) 4. *Hyperdynamic circulation* and consequently high output HF 5. Enhanced *hypoxic pulmonary vasoconstriction*	

Our case had high output HF, as confirmed by an echocardiogram. A high output HF is observed in many conditions, including chronic anemia, sepsis, obesity, hypercapnia, hyperthyroidism, and beriberi; notably, many of these pathologies can be corrected, potentially leading to the resolution of HF [4]. In severe anemia, the reduced oxygen-carrying capacity of blood triggers hemodynamic and non-hemodynamic compensatory responses; those physiologic adjustments consequently cause high output HF and PH, both of which may resolve upon correction of anemia [5]. Chronic tissue hypoxemia leads to peripheral vasodilation to optimize oxygenation and tissue perfusion [6]. The lower blood viscosity and increased nitric oxide synthase activity, observed in anemia, cause peripheral vasodilation with reduced systemic vascular resistance that triggers neurohormonal activation with salt and fluid retention, resulting in high output HF with hyperdynamic circulation [7].

Iron deficiency alone has a recognized association with HF, and it is linked to symptoms, exercise capacity, and poor survival, while iron transfusion is linked with improved exercise capacity and clinical well-being [8]. Iron plays a role in the normal activity of the citric acid cycle and reactive oxygen species, thus iron deficiency may intensify local myocardial oxidative stress, causing myocardial damage, while correction of iron deficiency remarkably leads to the reversibility of LV dysfunction [9].

In addition to HF, our case was complicated by severe PH. Tissue hypoxemia in severe anemia evokes hypoxic pulmonary vasoconstriction (HPV) and subsequently pulmonary hypertension and right heart failure [10]. HPV is a protective physiological reflex that optimizes ventilation/perfusion matching, thus improving systemic oxygen delivery [11]. Iron deficiency, even in the absence of anemia, enhances HPV response, as demonstrated in a randomized trial by Smith and colleagues in which HPV was attenuated by iron supplementation and exacerbated by iron depletion [12].

In our case, an echocardiogram confirmed a small pericardial effusion that responded well to the correction of anemia. Pericardial effusions are associated with numerous pathologies, including cardiac failure, pulmonary hypertension, neoplastic, infections, and idiopathic processes. However, pericardial effusions are rarely observed in the setting of chronic severe IDA and literature remains sparse, with only two cases of pericardial effusion reported [13]. Pericardial effusions are common (25–30%) in pulmonary arterial hypertension (PAH) but tend to be small volume and rarely of hemodynamic significance, as in our case [14]. Pericardial effusion, in our case, likely resulted from PH, causing raised right-sided filling pressures leading to increased filtration and lymphatic obstruction, resulting in transudative pericardial effusion [15].

Management of reduced ejection fraction heart failure (HF) is well supported by many randomized clinical trials and published guidelines; on the contrary, the evidence base for the management of high output HF is scarce and lacks the support of international guidelines [16]. The use of conventional vasodilators, such as angiotensin receptor blockers (ARB), angiotensin-converting enzyme inhibitors (ACEi), and β-blockers with vasodilatory properties (for example, carvedilol and nebivolol), may further aggravate lower systemic vascular resistance observed in high output HF, causing deterioration in clinical status, and are therefore not recommended [17]. Management in such cases should be targeted at correcting anemia, along with use of diuretics and dietary restriction of salt and water. While cautionary blood transfusion at Hb ≤ 7 may be necessary, vigorous volume loading may exacerbate the congestive state and pulmonary edema and thus should be avoided [18].

The differential diagnosis for HF, PH, and pericardial effusion is wide and we carefully excluded other possible causes by carrying out appropriate investigations. Our management was focused on the underlying etiology of the case, that is, anemia, rather than its complications. We reversed the iron deficiency anemia with blood transfusion and iron replacement with excellent clinical recovery. A repeat echocardiogram confirmed remarkable improvements in cardiac parameters, pericardial effusion, and pulmonary pressures.

## Conclusions

While iron deficiency anemia complicated by pericardial effusion is rare, we report a presentation of chronic IDA complicated by cardiac failure, pulmonary hypertension, and pericardial effusion. To our knowledge, this is a unique presentation of IDA, not yet reported in the literature. Our case highlights the fact that HF, PH, and pericardial effusion complicating IDA are the results of internal homeostatic mechanism, and thus it is important to target the underlying etiology rather than the complications. Our case report supports the association of IDA with HF and PH and their reversibility on the correction of anemia. Further research is needed to confirm if these complications are completely reversed in cases of chronic severe anemia.

## Data Availability

Not applicable.

## Abbreviations

HF: Heart failure
IDA: Iron deficiency Anemia
PH: Pulmonary hypertension
